# Outcomes of Infants Born at 21 Weeks’ Gestational Age

**DOI:** 10.1001/jamanetworkopen.2025.48211

**Published:** 2025-12-12

**Authors:** Rachael M. Hyland, Hatice Dilara Mat, Timothy J. Boly, Brady J. Thomas, Amy H. Stanford, Heidi M. Harmon, Jennifer R. Bermick, Rebecca Chuffo Davila, Tarah T. Colaizy, John M. Dagle, Jonathan M. Klein, Andrea L. Greiner, Edward F. Bell, Patrick J. McNamara

**Affiliations:** 1 Division of Neonatology, Department of Pediatrics, University of Iowa, Iowa City; 2 Division of Maternal-Fetal Medicine, Department of Obstetrics & Gynecology, University of Iowa, Iowa City

## Abstract

**Importance:**

Advances in medical care and clinician experience have lowered the gestational age at which resuscitation is considered for extremely premature infants. Some centers now offer trials of resuscitation at 21 weeks’ gestational age. Minimal data are available to guide this practice.

**Objective:**

To describe morbidities and outcomes of resuscitated infants at 21 weeks’ gestational age.

**Design, Setting, and Participants:**

This retrospective case series included all infants born at 21 weeks’ gestational age in a single-center level IV academic neonatal intensive care unit (NICU) at the University of Iowa from January 1, 2010, through February 28, 2025. Data were analyzed from April 1 to August 15, 2025.

**Exposure:**

Extremely preterm birth at a gestational age of 21 weeks.

**Main Outcomes and Measures:**

Demographic characteristics, therapies, morbidities, and test results were collected throughout hospitalizations from electronic medical records for extremely premature infants who received any attempted resuscitation and their mothers, including hourly cardiorespiratory data for the first 72 hours for infants admitted to the NICU. Postdischarge outcomes through the end of the study period were collected as available.

**Results:**

Twenty-two infants were born alive at 21 weeks’ gestational age during the study period, and an additional 230 were stillborn. Resuscitation was attempted in 17 (77%) of the infants born alive; of these, 6 (35%) survived to discharge from the NICU (earliest in 2021; youngest, 21 weeks, 0 days; birth weight, 285-430 g), 1 (6%) remained hospitalized, and 10 (59%) died. All births were inborn vaginal deliveries. Infants who survived (including the infant who remained hospitalized) were less likely part of a multiple gestation compared with those who did not (1 of 7 [14%] vs 6 of 10 [60%]) and more likely exposed to a complete course of antenatal corticosteroids (3 of 7 [43%] vs 0 of 10). Most of the infants who survived had early cardiorespiratory instability treated with vasoactive medications and/or inhaled nitric oxide (5 [71%]). Three (43%) had no or grade 1 intraventricular hemorrhage (IVH), 2 (29%) had grade 2 IVH, and 2 had severe (grade 3) IVH (29%); none required neurosurgical intervention. The 6 infants who were discharged received low-flow supplemental oxygen, and none required tracheostomy. Rates of resuscitation increased during the study period.

**Conclusions and Relevance:**

In this case series of infants born alive at 21 weeks’ gestational age, selective resuscitation resulted in 35% survival to discharge, in some cases with minimal morbidities. These descriptive data may prompt prospective study and reporting at this gestational age. Long-term outcome data are needed.

## Key Points

**Question:**

What are the outcomes of infants born alive at 21 weeks’ gestational age in a center that attempts resuscitation in selected cases?

**Findings:**

In this case series of 22 infants born alive at 21 weeks’ gestational age, resuscitation was attempted in 17 infants during the study period. Of these, 6 (35%) survived to discharge, and none required tracheostomy or neurosurgical intervention.

**Meaning:**

These findings suggest that some infants born at 21 weeks’ gestational age can survive, some with minimal morbidities, and that prospective study and outcome reporting may be warranted in this population.

## Teaser

This case series presents survival and morbidity outcomes for infants born alive and resuscitated at 21 weeks’ gestational age at a center that attempts resuscitation in selected cases.

## Introduction

The earliest gestational age at which trials of neonatal resuscitation are offered continues to decrease with advances in medical care and as experience in individual centers evolves. Reported outcomes in large cohort studies in the US and internationally include infants as premature as 22 weeks’ gestational age.^1,2,3,4,5,6^ Data are not yet available at 21 weeks’ gestational age outside rare case reports.^7,8,9,10^ There are marked between-institution differences in survival, morbidity, and rates of attempted resuscitation at the early extremes of gestational age.^11,12^

Survival to discharge in the earliest currently reported 22-week cohort, previously thought to be previable, has increased over time.^13,14^ The latest US cohort data, collected from 2013 to 2018 and again from 2020 to 2022, report survival rates of 30% to 35% for infants born at 22 weeks’ gestational age receiving resuscitation, improved from 23% survival in a similar population from 2006 to 2011.^5,11,13^ There is considerable variability in survival at this gestational age, however, with reported survival as low as zero and as high as 83% within international and single-center cohorts.^6,11,15,16,17,18,19^ Data from our own center at the University of Iowa reported to the Vermont Oxford Network from 2014 to 2024 at 22 weeks’ gestational age show 66% survival to discharge (41 of 62 patients) among those resuscitated (eFigure in Supplement 1).

Risk of short- and long-term physical and intellectual morbidities are increased in extremely premature infants as well, but rates of morbidity, like survival data, are highly variable depending on the source, population, and time point.^13,20,21,22^ US cohorts born at 22 weeks’ gestational age and followed up at 18 to 22 months report a wide-ranging 39% to 55% with no or mild impairment and 18% to 33% with severe impairment.^11,13,23^

Reporting is further complicated by inconsistent definitions in the literature for neurodevelopmental outcomes and the limited availability of long-term outcome data beyond early childhood.^24^ Even with clear definitions of a given morbidity, there can be striking differences in the perception of what is a positive outcome between families and clinicians.^25,26,27,28^ Despite the limitations of these studies, outcome data remain a critical component of antenatal counseling and shared decision-making among parents, neonatologists, and obstetrical clinicians.^26,29,30^

Due in part to the large variability in neonatal outcomes and morbidity, the approach to periviable obstetrical intervention and neonatal resuscitation varies widely among institutions and clinicians.^11,31,32^ Resuscitation at less than 22 weeks’ gestational age has been historically rare, but in some centers, including the University of Iowa, resuscitation at 21 weeks is now offered in selected cases. In this retrospective case series, we present survival and morbidity outcomes for infants born alive and resuscitated at 21 weeks gestational age at the University of Iowa.

## Methods

All infants born alive at a gestational age of 21 weeks 0 to 6 days between January 1, 2010, and February 28, 2025, at the University of Iowa were included. Our center’s institutional review board approved the study with a waiver of consent due to the retrospective design, minimal risk to participants, and use of deidentified data to ensure patient privacy; the study adhered to the reporting guideline for case series. Participants were identified by an internal live birth registry, cross-referenced to our centers’ Neonatal Research Network Generic Database prospectively collected registry.^33^ Live birth was defined as any evidence of activity or heart rate present. Resuscitation was broadly defined as any attempt to revive or stimulate; this did not require the presence of a neonatologist or placement of a breathing tube. Neonatal intensive care unit (NICU) admission was defined as admission to the NICU for any period.

The systematic approach to care of extremely preterm infants at the University of Iowa has been described previously.^23,34,35,36,37,38^ Many of these strategies for care at 22 and 23 weeks’ gestational age have been extrapolated to our infants born at 21 weeks. A standardized approach to cardiovascular care was followed after launching a formal neonatal hemodynamics and targeted neonatal echocardiography (TNE) program in 2019.^39^

Initial counseling in periviable cases was between the obstetric clinician and pregnant person, after which neonatal staff were invited to a joint discussion if desired by the family. Antenatal counseling then included shared decision-making between obstetric and neonatal staff and the parents, referencing shared internal guidelines, with discussion that outcomes at this gestational age are unknown, that ability to resuscitate may be limited by the size of the patient and our equipment, and that there are unknown short- and long-term maternal risks. Resuscitation was not offered at 21 weeks if there were significant congenital anomalies and was not recommended in multiplicity greater than twin gestation (although it has occurred). If resuscitation was not desired by the family, this was fully supported, and comfort-directed care without resuscitation was provided. If there was no heart rate response with effective positive-pressure ventilation through an endotracheal tube, further resuscitation (eg, chest compressions, epinephrine) was not recommended but individualized. The decision to provide antenatal corticosteroids was made by the obstetric team based on maternal and fetal considerations.

Neonatal and maternal demographic characteristics, treatments, diagnoses, and laboratory results were abstracted from the electronic medical record for all resuscitated infants. Dating of gestational age was obtained by best obstetric estimate, including last menstrual period, fetal ultrasonography, and in vitro fertilization records.

Early clinical course outcomes were abstracted from electronic medical records and reported for all infants admitted to the NICU. Data were abstracted every 2 hours for the first 72 hours for these infants, including ventilatory settings, blood gas levels, blood pressure (BP), and therapies with specific doses of any vasopressor, inotrope, and inhaled nitric oxide (iNO). Respiratory severity scores (RSS) and vasoactive-inotropic scores were calculated; higher scores are associated with increased morbidity and mortality.^40,41^ Detailed morbidity data throughout the hospitalizations were abstracted and reported.

### Statistical Analysis

Data were analyzed from April 1, 2025, to August 15, 2025. Descriptive statistical analyses were performed using SPSS Statistics for Windows, version 28.0.1.1 (IBM Corporation). In the case of missing or incomplete data, estimates were excluded from calculations. Due to the small sample size, statistics comparing infants who survived with those who died were not performed.

## Results

### Demographic Data

**Figure 1.**
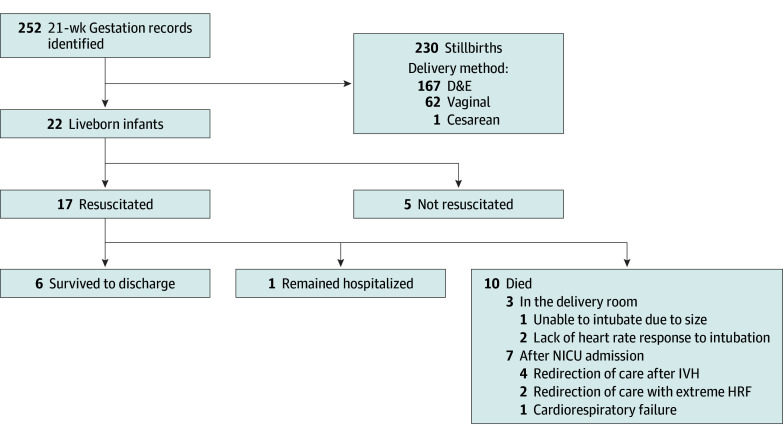
Outcomes at 21 Weeks’ Gestational Age Data are from the University of Iowa, January 2010 through February 2025. D&E indicates dilation and evacuation; HRF, hypoxemic respiratory failure; IVH, intraventricular hemorrhage; NICU, neonatal intensive care unit.

During the study period, there were 22 infants born alive at 21 weeks’ gestational age. There were an additional 230 fetuses at 21 weeks classified as stillbirths during the period. Seventeen of the 22 liveborn infants (77%) were resuscitated (8 female [47%] and 9 male [53%]; median age, 21 weeks 5 days [range, 21 weeks 0 days to 21 weeks 6 days]) and were included in the analysis. Of these, 6 (35%) were discharged home from the NICU, 1 (6%) remained hospitalized at the end of the study, and 10 (59%) died (3 in the delivery room, 7 in the NICU) (Figure 1).

**Figure 2.**
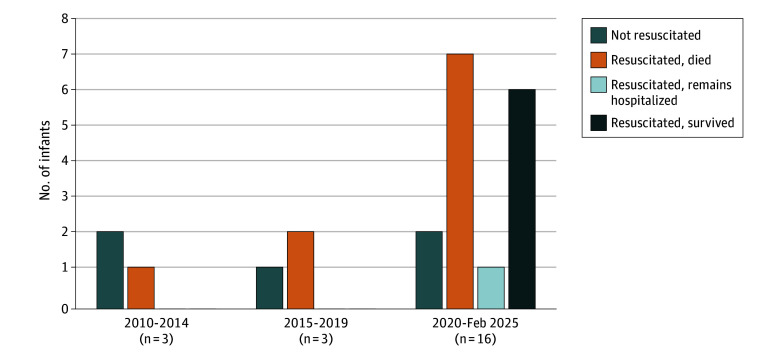
Use of Resuscitation Over Time of Infants Born Alive at 21 Weeks’ Gestational Age

Rates of resuscitation and NICU admission increased throughout the study period (Figure 2). During the first 10 years (2010-2019), 6 infants were liveborn, 3 (50%) of whom underwent resuscitation. None of these infants survived. In the next 5 years (2020 to February 2025), 16 infants were liveborn, 14 (88%) of whom underwent resuscitation.

Neonatal and maternal demographics for the 17 resuscitated infants are shown in Table 1. Birth weights ranged from 250 to 450 g, 7 patients (41%) were small for gestational age (extrapolated data, PediTools^42^), and 7 (41%) were part of a multiple gestation (including 1 set of dichorionic-triamniotic triplets and 2 pairs of monochorionic-diamniotic twins).

**Table 1.** Demographic Characteristics of Mothers and Infants Born Alive at 21 Weeks’ Gestational Age Receiving Active Resuscitation

Characteristic	No. (%)		
	Infant survived (n = 7)^a^	Infant died (n = 10)	All infants (N = 17)
**Infant**			
Gestational age, median (range), wk^b^	21^4^ (21^0^-21^6^)	21^5^ (21^0^-21^6^)	21^5^ (21^0^-21^6^)
Birth weight, mean (SD) [range], g^c^	372 (56) [285-430]	368 (68) [250-450]	370 (61) [250-450]
SGA	3 (43)	4 (40)	7 (41)
LGA	0	0	0
Fetal growth restriction	1 (14)	3 (30)	4 (24)
Multiple gestation	1 (14)	6 (60)	7 (41)
Sex			
Female	3 (43)	5 (50)	8 (47)
Male	4 (57)	5 (50)	9 (53)
Admitted to NICU	7 (100)	7 (70)^f^	14 (82)
Discharged from NICU	6 (86)	0	6 (35)
Age at death, median (IQR), d	NA	1 (0-33)	NA
Antenatal corticosteroids^d^			
None	2 (29)	2 (20)	4 (24)
Partial course	2 (29)	8 (80)	10 (59)
Complete course	3 (43)	0	3 (18)
Rupture of membranes			
>18 h^e^	5 (71)	2 (25)	7 (47)
>7 d	0	1 (10)	1 (6)
Inborn	7 (100)	10 (100)	17 (100)
Vaginal delivery	7 (100)	10 (100)	17 (100)
Breech birth	4 (57)	3 (30)	7 (41)
Apgar scores, median (IQR)			
1 min	1.0 (1.0-2.0)	2.0 (1.0-4.0)	1.0 (1.0-2.5)
5 min	4.0 (1.0-7.0)	3.0 (3.0-6.0)	3.0 (1.0-6.0)
Delayed cord clamping performed	3 (43)	2 (20)	5 (29)
No. of intubation attempts in delivery room, median (IQR)	2 (1-2)	2 (1-3)	2 (1-3)
Advanced delivery room resuscitation	1 (14)	1 (10)	2 (12)
**Maternal** ^f^			
Advanced maternal age	3 (43)	0	3 (23)
Complete antenatal care	6 (86)	7 (100)	12 (92)
Obesity	5 (71)	2 (29)	6 (46)
SSRI use	1 (14)	2 (29)	2 (15)
Substance or smoking use	0	2 (29)	2 (15)
Intra-amniotic infection	2 (29)	4 (57)	6 (46)
Gestational diabetes	3 (43)	1 (14)	4 (31)
Gestational hypertension	2 (29)	1 (14)	3 (23)
Preeclampsia	1 (14)	0	1 (8)

Abbreviations: LGA, large for gestational age; NA, not applicable; NICU, neonatal intensive care unit; SGA, small for gestational age; SSRI, selective serotonin reuptake inhibitor.

^a^ One patient remained admitted at the time of manuscript submission (postmenstrual age, >40 wk).

^b^ Fifteen infants were dated by best obstetrical estimate with last menstural period (LMP) and first-trimester ultrasonography; 1, in vitro fertilization; and 1, LMP with 13-week ultrasonography. Superscript numbers indicate No. of days.

^c^ One infant who did not survive had an estimated weight only that was not included in mean calculations (estimated weight, 150-200 g); weight at autopsy was 110 g.

^d^ The course was designated as complete if at least 2 doses of betamethasone were given, with the second dose 24 hours or more prior to delivery. Partial dosing reflected any betamethasone given not meeting these criteria, including dosing immediately prior to delivery.

^e^ Not available for 2 infants (estimated 2 days but not definitive) and not included.

^f^ Dataset included 2 sets of monochorionic-diamniotic twins and 1 dichorionic-triamniotic triplet gestation, with 13 mothers in total and 17 infants. One of the 4 patients in the monochorionic-diamniotic twin gestations survived (1 in each pair was admitted to the NICU), and none of the triplets survived (2 were admitted to the NICU). One mother was therefore included in both “survived” and “died” outcomes.

Survivors were less likely to be part of a multiple gestation (1 of 7 [14%] vs 6 of 10 [60%]). Survivors were more likely to have received a complete course of antenatal steroids (3 of 7 [43%] vs 0); however, similar numbers of survivors and nonsurvivors received no antenatal corticosteroids (2 of 7 [29%] and 2 of 10 [20%]). One- and 5-minute Apgar scores were low for all patients, with median scores of 1.0 (IQR, 1.0-2.5) and 3.0 (IQR, 1.0-1.6), respectively. Maternal characteristics and morbidity were similar between survivors and nonsurvivors, including frequent preterm prolonged rupture of membranes (7 of 15 [47%] of the cohort with data available).

All patients underwent inborn vaginal delivery, 7 (41%) were breech, and only 5 (29%) received limited delayed cord clamping (15-30 seconds in duration). Intubation attempts were performed by senior neonatology fellows or staff. Success at first attempt occurred in 6 (35%) of the 17 patients; multiple attempts were required in 11 (65%). Successful intubation took a median of 2 attempts (range, 1-4) and was unsuccessful in 1 patient due to the mouth not accommodating the laryngoscope.

### Early Clinical Course Data

Table 2 presents details of the clinical course during the first 72 postnatal hours for the 14 infants who were admitted to the NICU. Time from birth to NICU admission was 20 minutes or less for all patients except 1 who required extensive resuscitation in the delivery room (eMethods in Supplement 1). Central umbilical access was attempted in all patients; umbilical arterial catheter access was achieved in 8 patients (57%) and central umbilical venous placement was achieved in 13 (93%). Surfactant (poractant alfa) was given to all patients after endotracheal tube position was confirmed on chest x-ray at a mean of 1.4 (SD 0.4) postnatal hours (within 1 hour in only 1 patient).

**Table 2.** Early Clinical Course (First 72 Hours) Outcomes for Infants Admitted to the NICU

Outcome	Infants, No. (%)		
	Survived (n = 7)^a^	Died (n = 7)	All (n = 14)
Time from delivery to NICU admission, mean (SD), min	17.8 (4.2)	17.1 (2.0)	17.5 (3.2)
Central UAC obtained	5 (71)	3 (43)	8 (57)
Central UVC obtained	6 (86)	7 (100)	13 (93)
Age at first surfactant dose, mean (SD), postnatal h	1.4 (0.1)	1.4 (0.6)	1.4 (0.4)
No. of surfactant doses, median (IQR)	2 (1-2)	2 (1-2)	2 (1-2)
RSS, mean (SD) [range]^b^	2.9 (1.4) [1.3-9.5]	4.8 (2.5) [1.7-11.4]	3.6 (2.1) [1.3-11.4]
Negative blood culture at birth	7 (100)	7 (100)	14 (100)
No. of TNE examinations performed during first 72 h, median (IQR)	3.0 (2.0-5.0)	0 (0-3.0)	2.5 (0-4.3)
Patient received a TNE^c^	7 (100)	3 (43)	10 (71)
Age at first TNE (if applicable), mean (SD), h	3.4 (2.3)	7.9 (5.5)	4.7 (3.9)
Acute PH on any TNE	6 (86)	3 (100)	9 (90)
Cardiac dysfunction on any TNE	2 (29)	2 (67)	4 (40)
PDA on any TNE	7 (100)	3 (100)	10 (100)
Hydrocortisone therapy	7 (100)	7 (100)	14 (100)
Age at first hydrocortisone dose, median (IQR) [absolute range], h	2.2 (6.1) [1.3-50.3]	2.6 (1.6) [1.3-4.2]	2.4 (2.1) [1.3-50.3]
Hypotension	5 (71)	7 (100)	12 (86)
SBP <28 mm Hg	3 (43)	4 (57)	7 (50)
DBP <12 mm Hg	2 (29)	6 (86)	8 (57)
hsPDA treatment	4 (57)	1 (14)	5 (36)
iNO treatment	3 (43)	6 (86)	9 (64)
Maximum iNO dose, ppm	5	20	NA
Duration of iNO therapy until discontinued, mean (SD) [absolute range], h	18.7 (4.9) [13-22]	NA^d^	NA
Vasopressor infusion^e^	2 (29)	3 (43)	5 (36)
Inotrope infusion^f^	4 (57)	5 (71)	9 (64)
VIS, mean (SD) [absolute range]^g^	0.81 (2.0) [0-8]	2.1 (4.6) [0-25]	1.3 (3.2) [0-25]
SBP (every 2 h for 72 h), mean (SD), mm Hg	37.3 (6.1)	38.3 (9.0)	37.6 (7.2)
DBP (every 2 h for 72 h), mean (SD), mm Hg	26.1 (6.5)	20.7 (6.5)	24.4 (7.0)
HUS performed in the first 72 h	7 (100)	4 (57)	11 (79)
None	3 (43)	0	3 (27)
Low grade IVH (grade 1-2)	3 (43)	2 (50)	5 (45)
High grade IVH (grade 3-4)	1 (14)	2 (50)	3 (27)

Abbreviations: DBP, diastolic blood pressure; hsPDA, hemodynamically significant patent ductus arteriosus; HUS, head ultrasonography; iNO, inhaled nitric oxide; IVH, intraventricular hemorrhage; NICU, neonatal intensive care unit; PDA, patent ductus arteriosus; PH, pulmonary hypertension; RSS, respiratory severity score; SBP, systolic blood pressure; TNE, targeted neonatal echocardiography; UAC, umbilical arterial catheter; UVC, umbilical venous catheter; VIS, vasopressor-inotropic score.

^a^ One patient included remained admitted at the time of manuscript submission (postmenstrual age, >40 weeks).

^b^ Calculated as mean airway pressure × fraction of inspired oxygen.

^c^ Available after 2019. Two patients admitted to the NICU were born prior to this, when cardiology echocardiography was available instead but was not obtained. Two patients in the post-TNE era died before assessment could be completed.

^d^ Not discontinued in this group (continued until death; range, 2-121 hours).

^e^ Vasopressors included dopamine (n = 1), norepinephrine (n = 4), and vasopressin (n = 1). Two infusions (norepinephrine and vasopressin) were given in 1 patient.

^f^ Inotropes included dopamine (n = 1), dobutamine (n = 5), epinephrine (n = 4). Two therapies (dobutamine, epinephrine) in 1 patient.

^g^ Calculated as dopamine dose (µg/kg/min) plus dobutamine dose (µg/kg/min) plus [100 × epinephrine dose (µg/kg/min)] + 10 × milrinone dose (µg/kg/min)] + [10 × vasopressin dose (mU/kg/min)] + [100 × norepinephrine dose (µg/kg/min)].

On admission to the NICU, all patients were connected to high-frequency jet ventilator support according to our center’s standard respiratory practice.^35^ RSS varied widely during the first 72 hours from 1.3 to 11.4, with RSS being lower in infants who survived (mean [SD], 2.9 [1.4] vs 4.8 [2.5]). An initial high oxygen requirement was common (mean [SD] fraction of inspired oxygen [Fio_2_] on admission, 0.92 [0.17]); however, the mean (SD) Fio_2_ during the first 72 hours was only 0.47 (0.22) (eTable in Supplement 1).

Early hemodynamic instability was evident with frequent vasopressor (5 [36%]), inotrope (9 [64%]), and/or iNO (9 [64%]) therapy during the first 72 hours. Twelve of the infants in the NICU cohort (86%) received 1 or more therapies (5 of 7 survivors [71%]; 7 of 7 nonsurvivors [100%]) during this early period. The highest vasopressor-inotropic score was 8.0 in survivors and 25.0 in nonsurvivors, with considerable variance during the 72 hours (variance, 3.8 in survivors and 21.2 in nonsurvivors). The maximum iNO dose in survivors was 5 ppm with a mean (SD) duration of 18.7 (4.9) hours, compared with 20 ppm in nonsurvivors (not discontinued).

All surviving patients had TNE performed within the first 12 hours, most (6 [86%]) in the first 4 postnatal hours, while only 3 nonsurvivors (43%) had TNE or cardiac echocardiography performed. Among these 10 patients who underwent TNE, cardiovascular phenotypes identified included acute pulmonary hypertension (PH) (9 [90%]), cardiac dysfunction (4 [40%]), and hemodynamically significant patent ductus arteriosus (hsPDA) (5 [50%]), with many infants (9 [90%]) experiencing multiple or transitioning phenotypes over this period.

Early mean BP measurements are shown in Table 2, with wide individual patient absolute ranges (eTable in Supplement 1) across the 72-hour time period, but lower variance in survivors; survivor systolic blood pressure variance was 7.7 mm Hg and diastolic BP variance was 9.2 mm Hg, compared with 24.5 and 16.6 mm Hg, respectively, in nonsurvivors. Hypotension occurred, at least transiently, in 12 infants (86%) (eMethods in Supplement 1). All patients were treated with hydrocortisone for presumed adrenal insufficiency and/or relative systolic or diastolic hypotension.

The 7 nonsurviving patients admitted to the NICU died on postnatal days 0 to 6. In all cases, the cause of death was listed as extreme prematurity, with complicating factors including respiratory failure in all, and in some pulmonary hemorrhage (n = 2) and IVH (n = 4). In 4 cases, care was redirected to comfort focused after finding IVH in the setting of significant clinical instability. Care for 2 patients was redirected after prolonged desaturations at 3 and 4 hours of age (prior to head imaging). One patient died after cardiorespiratory failure with refractory hypoglycemia despite code resuscitation on postnatal day 3 (prior to head imaging).

### Outcome Data

Available outcome data are provided in Table 3 for the 6 surviving infants and the 1 infant who remained hospitalized at submission (eMethods in Supplement 1). All infants were extubated directly from high-frequency jet ventilator support to noninvasive neurally adjusted ventilatory assistance delivered via nasopharyngeal tube. Successful extubation occurred at a median age of 79.5 (IQR, 61.8-89.3) days (approximately 32 weeks’ postmenstrual age). Grade 3 bronchopulmonary dysplasia (BPD) at postmenstrual age of 36 weeks occurred in only 1 patient, with the remainder having grade 2 BPD. No infant required tracheostomy, and the 6 discharged home did so receiving low-flow nasal cannula oxygen therapy.

**Table 3.** Clinical Outcomes for Surviving Infants^a^

Outcome	Median (IQR)	Age range, d (PMA range, wk)^b^
Age at discharge (n = 6), median (IQR), d	182.5 (169.0-202.5)	151-213 (43^0^-52^2^)
Respiratory outcomes		
Age at first extubation, median (IQR), d	60 (45-76)	40-83 (27^1^-33^5^)
First extubation successful, No. (%)	3 (43)	NA
Age at successful extubation, median (IQR), d	79.5 (61.8-89.3)	58-102 (30^1^-36^3^)
IMV duration, median (IQR), d	76.0 (63.0-83.0)	58-85 (NA)
NIPV duration, median (IQR), d	87.5 (65.8-127.0)	65-166 (NA)
Age at transition to low-flow oxygen, median (IQR), d	157.5 (140.3-176.8)	129-185 (39^6^-48^0^)
BPD at 36 weeks – Jensen criteria, No. (%)	7 (100)	NA
No BPD	0	NA
Grade 1	0	NA
Grade 2	6 (86)	NA
Grade 3	1 (14)	NA
Tracheostomy, No. (%)	0	NA
Discharged on oxygen therapy, 0.5-2 LPM nasal cannula, No. (%)	6 (100)	NA
Cardiovascular outcomes		
hsPDA medical treatment, No. (%)	7 (100)	NA
Age at first hsPDA medical treatment, median (IQR), d	3 (2-4)	1-4 (21^1^- 22^3^)
hsPDA interventional treatment, No. (%)	5 (71)	NA
Age at hsPDA intervention, median (IQR), d	26.0 (22.0-55.5)	19-74 (24^4^-31^4^)
Systemic hypertension requiring treatment, No. (%)^c^	2 (29)	NA
Chronic pulmonary hypertension, No. (%)^d^	4 (57)	NA
Discharged on chronic PH therapy, No. (%)	2 (33)	NA
Gastrointestinal outcomes		
Age at first enteral feed, median (IQR), d	3.0 (2.0-10.0)	2-14 (21^6^-23^2^)
Age at full enteral feeds, median (IQR), d	45.0 (42.0-67.0)	33-160 (26^4^-43^6^)
Necrotizing enterocolitis, any stage, No. (%)	0	NA
Spontaneous intestinal perforation, No. (%)	1 (14)	NA
Discharged with oral ad lib feeding, No. (%)	2 (33)	NA
Discharged with oral and/or NG feeding, No. (%)	4 (67)	NA
Discharged with gastrostomy tube, No. (%)	2 (33)	NA
ROP diagnosis (any grade), No. (%)	7 (100)	NA
ROP stage ≥3, No. (%)	2 (29)	NA
ROP treatment, No. (%)	2 (29)	NA
Neurological outcomes		
Seizure, No. (%)	1 (14)	NA
Intraventricular hemorrhage (highest grade throughout hospitalization), No. (%)		
None	2 (29)	NA
Grade 1	1 (14)	NA
Grade 2	2 (29)	NA
Grade 3	2 (29)	NA
Grade 4	0	NA
Periventricular leukomalacia, No. (%)	1 (14)	NA
Cerebellar hemorrhage, No. (%)	2 (29)	NA
Severe ventriculomegaly at 36 wk PMA, No. (%)	0	NA
Neurological surgical intervention, No. (%)	0	NA
MRI prior to discharge, No. (%)	4 (67)	NA
Neurodevelopmental outcomes for infants >6 mo corrected age (n = 4), No. (%)		
Hearing impairment	1 (25)	NA
Visual impairment	1 (25)	NA
Cerebral palsy	2 (50)	NA
Development delay on last assessment	3 (75)	NA

Abbreviations: BPD, bronchopulmonary dysplasia; hsPDA, hemodynamically significant patent ductus arteriosus; IMV, invasive mechanical ventilation; LPM, liters per minute; NG, nasogastric; NIPV, noninvasive positive pressure ventilation; PH, pulmonary hypertension; PMA, postmenstrual age; ROP, retinopathy of prematurity.

^a^ One patient remained admitted at the time of manuscript submission and was post term-corrected, has been extubated to noninvasive respiratory support and their data are included when relevant to the category (percentiles demonstrate the correct number per category; if this patient had the complication they were included, otherwise it was assumed the complication may yet occur during the admission and not included in the number).

^b^ Superscript numbers indicate No. of days.

^c^ Indicates persistent systolic or diastolic blood pressure greater than the 95th percentile. Treatment was with enalapril in both cases.

^d^ Defined as persistent markers of elevated right ventricular systolic pressure 30 mm Hg or greater or systolic eccentricity index 1.3 or greater on consecutive targeted neonatal echocardiography studies at least 7 days apart.

There were no cases of necrotizing enterocolitis and 1 case of intestinal perforation secondary to meconium obstruction of prematurity.^43^ Four patients were discharged with supplemental feeding (2 via nasogastric tube, 2 via gastrostomy tube).

All surviving patients were treated medically for hsPDA, and 5 (71%) received interventional PDA closure (transcatheter closure in 3 patients, bedside surgical ligation in 2 patients). Four patients (57%) were diagnosed with chronic PH, 2 of whom were discharged home receiving chronic PH therapy with enteral sildenafil.

IVH occurred in 5 survivors (71%); however, severe IVH occurred in only 2 (29%; grade 3 in both cases). No neurosurgical interventions were needed. One patient (14%) had seizures during their NICU course, managed with antiseizure medication from which they were subsequently weaned. Two patients (29%) required retinopathy of prematurity treatment, both with 1 bevacizumab injection.

All discharged patients were followed up with serial developmental assessments. Developmental findings are limited by the early chronological age of our survivors; only 4 discharged patients were older than 6 months corrected age at the time of study completion. One patient was older than 2 years of corrected age, and their developmental assessment findings were normal for corrected age. The remaining 3 patients were delayed in their milestones but progressing; on their last developmental assessments they each scored borderline to high risk in the Bayley-4 screening test categories. Two of these patients were diagnosed with spastic-type cerebral palsy, and neither used orthotics, although 1 may in the future.^44^ One patient had mild to moderate hearing loss, and 1 patient had visual deficits requiring glasses. Three of the 6 patients discharged home required rehospitalization following their initial discharge for various reasons, including respiratory viral illness, inadequate weight gain, gastrostomy tube placement, and a supraglottoplasty for laryngomalacia diagnosed after discharge.

## Discussion

To our knowledge, there have been no previously published cohorts of survivors born at 21 weeks’ gestational age. Our case series data provide early experience within our center and a needed reference point as we build collective understanding of the challenges and possibilities facing these infants.

There are several notable patterns and observations found in our data. First, rates of resuscitation have increased significantly in a short period at our center (Figure 2). During the past 5 years, resuscitation rates increased to 87.5% and were 100% in the last year (2024). Careful consideration of resuscitation continues on a case-by-case basis.

Second, antenatal corticosteroids were given in most of these pregnancies, and among the survivors were the only 3 infants whose mothers completed courses of corticosteroids. Growing evidence suggests that antenatal corticosteroids offer a survival benefit at gestational ages of 22 and 23 weeks.^45,46^ This evidence recently prompted an updated American College of Obstetrics and Gynecology practice advisory to suggest consideration of antenatal corticosteroids at 22 weeks’ gestational age after appropriate counseling.^47,48^ The advisory continues to not recommend antenatal corticosteroids at 21 weeks’ gestational age due to lack of data; however, this is an area of active debate.^49^

Third, our experience suggests that the technical skills needed to support resuscitation and care at this gestational age are high. Despite high experience within our group in the care of extremely premature infants, there was frequent need for multiple intubation attempts in the delivery room (65%), a relatively low success rate (57%) of umbilical arterial catheter placement, and the first dose of surfactant therapy given by 1 hour after birth in only 1 patient. Patient size and clinician experience are both known contributors to procedural success, which are both compounded in the rare occurrence of resuscitation at 21 weeks’ gestational age.^50^ Maintaining periprocedural stability and warmth, balancing expediency, in this fragile group remains challenging.

Fourth, our first 72-hour hemodynamic data highlight the rapidly changing physiology that can be experienced in patients of this gestational age. Early Apgar scores were low, and vasoactive-inotropic scores and RSS varied widely. iNO was used frequently but judiciously; in our survivors the iNO maximum dose was 5 ppm and they were weaned off therapy in less than 24 hours.^51^ These observations highlight therapeutic trends toward responsive and physiology-based management and are endorsed by emerging data from our center and others that provide TNE-based hemodynamic care.^19,36,39,52,53^ Of note, there are leading centers in periviable care that do not use TNE, highlighting that a standardized and comprehensive approach to care is likely as important as or more important than a particular care strategy.^54,55^

Our data also show chronic cardiovascular patterns, with chronic PH affecting 57% of our cohort of surviving infants. BPD-associated PH is a known entity thought to affect approximately 25% of preterm infants with moderate to severe BPD, but emerging studies specific to extremely preterm infants show even higher rates, which align more closely with our findings.^56,57^ Our chronic respiratory data revealed encouragingly a low rate of severe BPD (1 case of grade 3) and no instances of tracheostomy, despite early respiratory instability and and median invasive mechanical ventilation duration of greater than 10 weeks (76 [63-83] days).

### Limitations

Our data are limited by small numbers and our single-center experience, minimizing extrapolation to other centers with variable management practices and patient populations. We attempted to include all liveborn infants with a gestational age of 21 weeks; however, we cannot be sure that every infant was formally assessed for a heart rate at birth, which could underestimate our denominator of liveborn infants. The number of reported liveborn infants increased through the study period for unclear reasons, which could be due to early underreporting or recognition prior to frequent resuscitations offered. We were able to accurately report the outcomes of all infants for whom resuscitation was attempted, so estimation of survival of actively treated infants is not prone to such bias. We are also limited by the possibility of incorrect dating, as only 1 patient was dated by in vitro fertilization. All infants in this cohort were born vaginally, which limits extrapolation to other obstetric delivery practices and may bias toward a decreased liveborn rate.

The ethical questions surrounding periviable resuscitation are complex and beyond the scope of this study. Recent editorials have urged caution when considering resuscitation of periviable infants and highlight the risk of infant and maternal morbidity, mortality, stress, and trauma related to prolonged hospitalizations and disproportionate use of resources.^31,58,59^ These considerations are a vital component of antenatal counseling and shared decision-making. However, also vital is the acknowledgment that death and disability are neither equal nor similar outcomes, and that morbidity outcomes are variably experienced and defined.^26,27,29^ Historic trends at each preceding gestational time point, and our data presented herein, suggest rates of resuscitation at 21 weeks’ gestational age will continue to increase. We suggest that consideration of resuscitation at 21 weeks is ethically permissible in experienced centers of excellence when there is shared decision-making between families and clinicians.

## Conclusions

In this case series of infants born alive at 21 weeks’ gestational age, previously believed to be previable, 35% of infants resuscitated survived to discharge from the NICU. There are no long-term outcomes yet available for this cohort. To understand the impact of our interventions and better counsel families, we encourage prospective data collection and reporting for both maternal and infant outcomes at this gestational age. We present our experience as a small step toward that aim, as additional data for neonatologists, obstetricians, and families to consider as they jointly make complex decisions in periviable patient care.

## Notes

Supplement 1. **eMethods.** Patient Characteristics and Treatment **eTable.** Supplemental Early Respiratory and Cardiovascular Clinical Course Details: First 72 Hours of Admission Data for Infants Admitted to the NICU **eFigure.** Active Resuscitation Survival Outcomes at 22 Weeks’ Gestational Age Across Multicenter and Single Sites, Compared With University of Iowa Outcomes at 22 and 21 Weeks’ Gestational Age

Supplement 2.
Nonauthor Collaborators

Supplement 3.
Data Sharing Statement
