# Error in Abstract

**DOI:** 10.1001/jamanetworkopen.2025.58314

**Published:** 2026-01-20

In the Original Investigation titled “Outcomes of Infants Born at 21 Weeks’ Gestational Age,”^1^ published December 12, 2025, the beginning date of data collection was incorrect in the abstract. This article has been corrected.
