# Supplementary material for: Outcomes of Infants Born at 21 Weeks’ Gestational Age
Source: JAMA Netw Open. 2025 Dec 12;8(12):e2548211. doi: 10.1001/jamanetworkopen.2025.48211 (PMC12701515; doi:10.1001/jamanetworkopen.2025.48211)
Supplement: Supplement 1. — eMethods. Patient Characteristics and Treatment eTable. Supplemental Early Respiratory and Cardiovascular Clinical Course Details: First 72 Hours of Admission Data for Infants Admitted to the NICU eFigure. Active Resuscitation Survival Outcomes at 22 Weeks’ Gestational Age Across Multicenter and Single Sites, Compared With University of Iowa Outcomes at 22 and 21 Weeks’ Gestational Age [file jamanetwopen-e2548211-s001.pdf]

Supplementary Online Content

Hyland RM, Mat HD, Boly TJ, et al; University of Iowa Neonatology Program. Outcomes of infants born at 21 weeks' gestational age. *JAMA Netw Open.* 2025;8(12):e2548211. doi:10.1001/jamanetworkopen.2025.48211

eMethods. Patient Characteristics and Treatment

eTable. Supplemental Early Respiratory and Cardiovascular Clinical Course Details: First 72 Hours of Admission Data for Infants Admitted to the NICU

eFigure. Active Resuscitation Survival Outcomes at 22 Weeks' Gestational Age Across Multicenter and Single Sites, Compared With University of Iowa Outcomes at 22 and 21 Weeks' Gestational Age

This supplementary material has been provided by the authors to give readers additional information about their work.

© 2025 Hyland RM et al. *JAMA Network Open*.

eMethods. Patient Characteristics and Treatment

Supplemental footnotes for Table 1 in the main text.

The majority (n=15) were dated by best obstetrical estimate with LMP and first-trimester ultrasound. One patient was dated by IVF, and one by LMP with 13-week ultrasound.

Birth weight was measured using an electronic bed scale on admission to the NICU (GE HealthCare Panda iRes Warmer, GE HealthCare Technologies, Chicago, IL, USA), which has a reported functional range down to 300 g and accuracy ± 10 g. Infants estimated to be less than 300 g had their weights measured by adding a pre-weighed fluid bag with the infant during the weight check and then subtracting the weight of the fluid bag from the determined infant weight. One infant who did not survive had an estimated weight only which was not included in mean calculations (estimated weight 150-200 g); their weight at autopsy was 110 g.

SGA and LGA designations were made from the PediTools electronic growth chart calculator, which uses extrapolated data to generate 21-week growth curves.

Fetal growth restriction defined as fetal weight or abdominal circumference less than the 10th percentile on fetal ultrasound.

Three patients died in the DR after attempted resuscitation, each were part of a separate multiple gestation. One patient, the smaller of the mono-di pair within a dichorionic-triamniotic triplet gestation, had an estimated fetal weight of 150-200 g and was too small for the 00 intubating blade to fit in the mouth (000 blade was not yet available at our institution). The remaining two patients were each a monochorionic-diamniotic twin from separate gestations, who required multiple intubation attempts (3 and 4 attempts) with unfortunately no heart rate response.

The longest duration of rupture was 12 days.

© 2025 Hyland RM et al. *JAMA Network Open*.

Delayed cord clamping was performed for only 15-30 seconds in all cases.

Advanced DR resuscitation was defined as the need for neonatal code medications and/or chest compressions according to the Neonatal Resuscitation Program. This occurred in two patients, sibling monochorionic-diamniotic twins, after parent request. One twin had heart rate improvement with intubation but then became bradycardic, and was treated with emergent UVC, saline bolus, and two doses of epinephrine with response. No chest compressions were performed. She ultimately survived to discharge. The second twin had unfortunately no respiratory effort and no heart rate response to intubation (3 attempts), and chest compressions were briefly performed at 9 minutes post-delivery while resuscitation medications were prepared. Resuscitation efforts were stopped after 45 seconds of compressions with no response.

Dataset included two sets of monochorionic-diamniotic twins and one dichorionic-triamniotic triplet gestation; 13 mothers in total and 17 infants. One of the four patients in the mono-di twin gestations survived (one in each pair was admitted to the NICU), and none of the triplets survived (two were admitted to the NICU). One mother was therefore included in both “survived” and “died” outcomes. There was no twin to twin or twin anemia polycythemia sequence diagnosed. One of the mono-di pair within the triplet gestation had selective fetal growth restriction.

Complete antenatal care was defined as at least 3 antenatal visits

Supplemental footnotes for Table 2 in the main text.

The time from delivery to admission relied on the first device data in the EMR, reflecting when the patient was connected to central monitors in the NICU.

© 2025 Hyland RM et al. *JAMA Network Open*.

One UVC not obtained centrally was utilized low-lying. UAC were considered central if on x-ray between thoracic (T) levels T6-10. Umbilical venous catheters (UVC) were considered central if at the level of the diaphragm T8-T9 on x-ray or on TNE assessment at the inferior vena cava and right atrium junction. One patient had an emergent UVC placed in the DR, this was replaced with a central UVC after admission to the NICU. One patient with an unsuccessful UAC had a peripheral arterial line placed on postnatal day 1 (26 gauge) which produced an arterial waveform but did not draw.

All early doses of surfactant were poractant alfa (Curosurf), repeat doses were either poractant alfa or calfactant (Infasurf).

Acute pulmonary hypertension (PH) was diagnosed by any of: (1) greater than 30% right-to-left shunting through the PDA; (2) right ventricular systolic pressure (RVSp) ≥ 30 mmHg on tricuspid regurgitant jet; or (3) evidence of septal flattening (systolic eccentricity index (sEI) ≥1.3) in the absence of profound hypotension.

Hypotension included systolic and/or diastolic hypotension obtained pre or post-ductally (via arterial or cuff pressures if arterial not available) defined as SBP < 28 mmHg and/or DBP < 12 mmHg, based on extrapolation from published metrics at older gestations and internal data.

Intraventricular hemorrhage (IVH) was defined by the Volpe classification system. All head ultrasounds during admission were reviewed independently by a radiologist and a member of the neonatal neurocritical care team and the higher grade (if discrepant) reading was reported.

Supplemental footnotes for Table 3 in the main text.

© 2025 Hyland RM et al. *JAMA Network Open*.

Bronchopulmonary dysplasia according to the Jensen criteria. Grade 1 nasal cannula less than or equal to 2 LPM, Grade 2 nasal cannula greater than 2 LPM or non-invasive positive pressure. Grade 3 invasive mechanical ventilation.

PDA interventional treatment was considered after medical treatment failure in a hemodynamically significant PDA (acetaminophen and 1 or 2 courses of indomethacin). This included 3 transcatheter closures and 2 bedside surgical ligations.

Chronic PH therapy was sildenafil for both infants

Necrotizing enterocolitis defined according to the modified Bell staging criteria

Spontaneous intestinal perforation secondary to inspissated meconium occured in one patient. This case required 10 cm of ileal resection and temporary ileostomy, which was successfully taken down with end-to-end anastomosis at 40 weeks PMA.

ROP therapy was with bevacizumab injection once for both patients (included the patient who remains admitted)

Intraventricular hemorrhage (IVH) was defined by the Volpe classification system. All head ultrasounds during admission were reviewed independently by a radiologist and a member of the neonatal neurocritical care team and the higher grade (if discrepant) reading was reported.

Severe ventriculomegaly defined as anterior horn width > 10 millimeters. Mild ventriculomegaly with anterior horn width 4-8mm (which did not require intervention) present in all 7 survivors

Neurodevelopmental outcomes for infants >6 months corrected age:

Mild to moderate hearing loss in one child on sedated ABR, repeated examination is pending.

Visual impairment occurred in one child who had 20/190 vision requiring glasses

Cerebral palsy diagnosis was based on General Motor Assessment (GMA) and Hammersmith Infant Neurological Examination which suggests high probably of CP in scores below 65. These

© 2025 Hyland RM et al. *JAMA Network Open*.

patients scored 51.5 and 49 and are both spastic-type. One child at 19 months' corrected age uses a Nimbo walker for independent steps and stands independently. The other child has asymmetric strength and mobility; at 14 months' corrected age she rolls each direction and sits unassisted with one point of contact.

The only child older than 2 years at time of submission has normal testing (Wechsler Preschool and Primary Scale of Intelligence-IV at 3 years of age IQ 86, Verbal Comprehension Index 89, Visual Spatial Index 89). Three younger children are all less than 18 months' corrected age and each scored High Risk or Borderline Risk on Bayley-4 screener in motor and cognitive sections.

© 2025 Hyland RM et al. *JAMA Network Open*.

eTable. Supplemental Early Respiratory and Cardiovascular Clinical Course Details: First 72 Hours of Admission Data for Infants Admitted to the NICU

	**Infant Survived** (n=7)	**Infant Died** (n=7)	**All Infants** (n=14)
**Respiratory Outcomes**			
HFJV on admission, No. (%)	7 (100)	7 (100)	14 (100)
FiO2 on admission, mean (SD) [absolute range]	0.86 (0.22) [0.40-1.0]	0.99 (0.04) [0.90-1.0]	0.92 (0.17) [0.40-1.0]
FiO2, 72 hours median (IQR)	0.37 (0.31-0.42)	0.56 (0.37-0.87)	0.39 (0.33-0.56)
FiO2, 72 hours mean (SD)	0.40 (0.16)	0.60 (0.25)	0.47 (0.22)
PEEP on admission, mean (SD) [absolute range], cm H2O	5.3 (0.8) [4-6]	5.2 (0.4) [5-6]	5.2 (0.6) [4-6]
PEEP, 72 hours median (IQR), cm H2O	5 (4-5)	5 (5-5)	5 (4-5)
PEEP 72 hours mean (SD), cm H2O	4.8 (0.6)	4.9 ± 0.9	4.8 ± 0.7
PiP on admission, mean (SD) [absolute range], cm H2O	25.9 (5.6) [18-35]	26.7 (4.6) [20-32]	26.2 (5.0) [18-35]
PiP, 72 hours median (IQR), cm H2O	27 (25-29)	33 (25-41)	27 (25-32)
PiP 72 hours mean (SD), cm H2O	27 (3.3)	33 (8.9)	29 (6.4)
Jet rate, on admission, median (IQR) [absolute range], bpm	300 [0] [300-360]	360 (60) [300-360]	300 (60) [300-360]
Jet rate, 72 hours median (IQR), bpm	300 (300-360)	300 (300-360)	300 (300-360)
Jet rate 72 hours mean (SD), bpm	317 (28)	325 (69)	320 (47)
MAP on admission, mean (SD), cm H2O	7.4 (1.3)	7.6 (0.8)	7.5 (1.1)
MAP, 72 hours median (IQR), cm H2O	7.2 (6.5-7.8)	7.4 (6.8-8.5)	7.2 (6.8-7.9)
MAP 72 hours mean (SD), cm H2O	7.3 (1.2)	7.9 (1.6)	7.5 (1.4)
pH on admission, mean (SD)	7.0 (0.09)	7.0 (0.2)	7.0 (0.15)
pH, 72 hours median (IQR)	7.31 (7.3-7.4)	7.27 (7.1-7.3)	7.30 (7.2-7.4)
pH 72 hours mean (SD)	7.3 (0.12)	7.25 (0.30)	7.28 (0.20)
pCO2 on admission, mean (SD), mm Hg	69 (17)	82 (31)	75 (25)
pCO2, median (IQR), mm Hg	49 (44-55)	55 (47-70)	51 (44-58)
pCO2 72 hours mean ± SD, mm Hg	50 (10.4)	59 (17.4)	53 (14)
**Cardiovascular Outcomes**			
SBP (every 2 h for 72 h), mean (SD) [absolute range], mm Hg	37.3 (6.2) [16-56]	38.3 (9.0) [6-62]	37.6 (7.2) [6-62]
DBP (every 2 h for 72 h), mean (SD) [absolute range], mm Hg	26.1 (6.5) [11-41]	20.7 (6.5) [3-43]	24.4 (7.0) [3-43]
24h SBP, mean ± SD	34.9 (6.0)	34.7 (10.5)	34.8 (7.9)
24h DBP, mean ± SD	24.7 (6.2)	19.0 (6.8)	22.6 (7.0)
48h SBP, mean ± SD	38.7 (6.0)	38.9 (6.1)	38.8 (6.0)
48h DBP, mean ± SD	28.3 (7.5)	21.6 (6.8)	26.2 (7.9)
72h SBP, mean ± SD	38.3 (5.8)	43.1 (6.6)	39.7 (6.4)
72h DBP, mean ± SD	25.5 (5.1)	22.2 (5.1)	24.5 (5.3)

© 2025 Hyland RM et al. *JAMA Network Open*.

eFigure. Active Resuscitation Survival Outcomes at 22 Weeks' Gestational Age Across Multicenter and Single Sites, Compared With University of Iowa Outcomes at 22 and 21 Weeks' Gestational Age

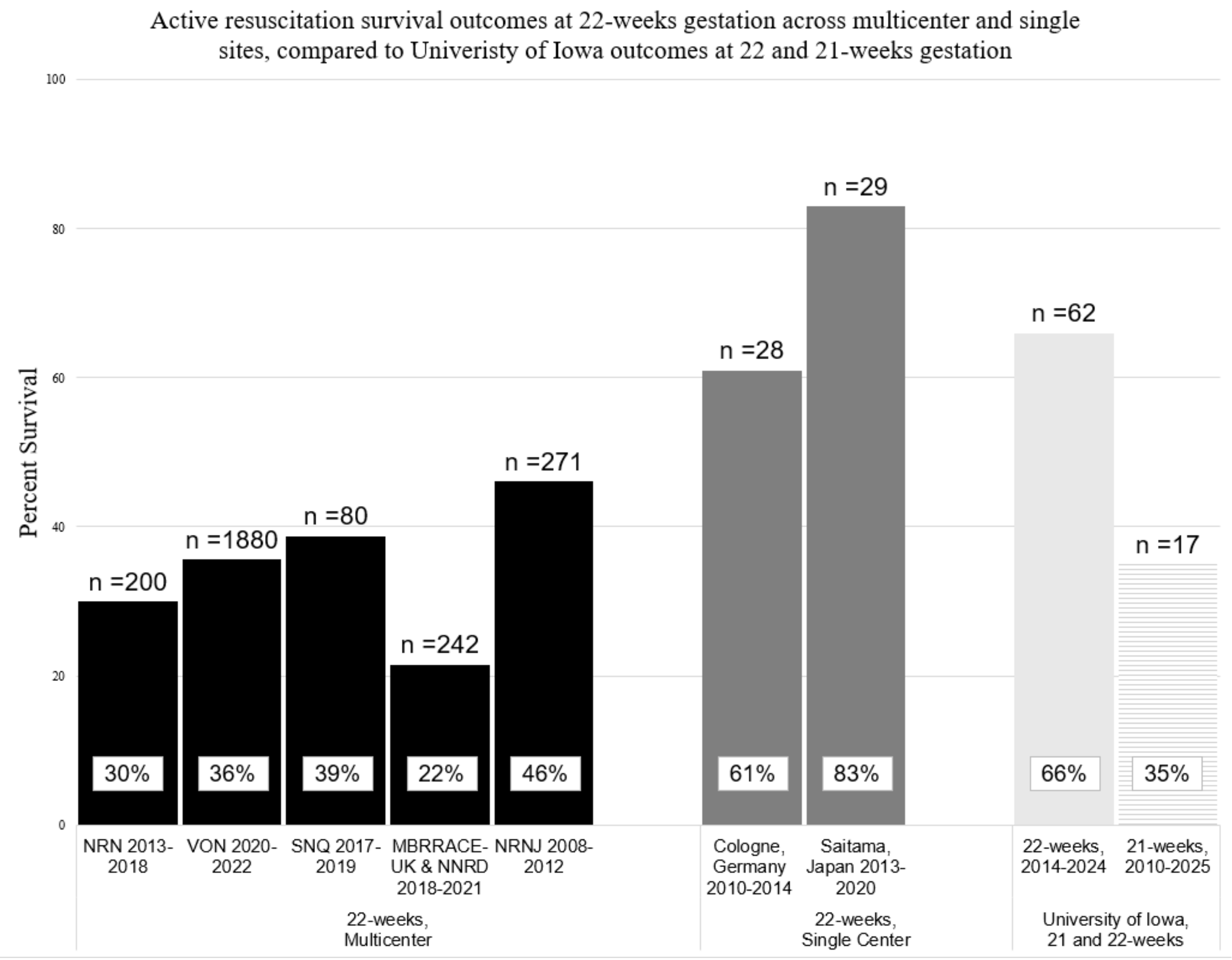

Referenced publications include

NRN: Neonatal research network, doi10.1001/jama.2021.23580; VON: Vermont oxford network, doi 10.1542/peds.2024-065963; SNQ: Sweden national registry, doi https://doi.org/10.1136/archdischild-2022-325164; MBBRACE-UK & NRRD: Mothers and babies: reducing risk through audits and confidential enquiries across the UK and national neonatal research network, doi 10.1136/bmjmed-2023-000579; NRNJ: Japan neonatal research network, doi 10.1136/bmjpo-2017-000211; Cologne, Germany doi 10.1038/s41372-023-01706-4. ; Saitama, Japan doi 10.1038/s41372-023-01706-4.

© 2025 Hyland RM et al. *JAMA Network Open*.
