# Supplementary material for: Outcomes of Infants Born at 21 Weeks’ Gestational Age
Source: JAMA Netw Open. 2025 Dec 12;8(12):e2548211. doi: 10.1001/jamanetworkopen.2025.48211 (PMC12701515; doi:10.1001/jamanetworkopen.2025.48211)
Supplement: Supplement 2. — Nonauthor Collaborators [file jamanetwopen-e2548211-s002.pdf]

Supplemental Online Content: Nonauthor Collaborators
*First name, last name, and suffix (if applicable) are required and will appear in PubMed.

*Group Name(s): University of Iowa Neonatology Program							
***First Name and Middle Initial(s)**	***Last Name**	***Suffix (eg, Jr, III)**	Academic Degrees	Institution	Location (city, state/province, country)	Role or Contribution, eg, chair, principal investigator	Group (if more than 1 Group listed in the byline) and/or Subgroup (eg, Steering Committee)
Aunum P.	Akhter		MD	University of Iowa Hospitals and Clinics	Iowa City, Iowa, US	Neonatologist	
Amy A.	Hobson		DO	University of Iowa Hospitals and Clinics	Iowa City, Iowa, US	Neonatologist	
Lindsey A.	Knake		MD	University of Iowa Hospitals and Clinics	Iowa City, Iowa, US	Neonatologist	
Stephanie S.	Lee		MD	University of Iowa Hospitals and Clinics	Iowa City, Iowa, US	Neonatologist	
Julie B.	Lindower		MD	University of Iowa Hospitals and Clinics	Iowa City, Iowa, US	Neonatologist	
Glenda K.	Rabe		MD	University of Iowa Hospitals and Clinics	Iowa City, Iowa, US	Neonatologist	
Adrianne R.	Bischoff		MD	University of Iowa Hospitals and Clinics	Iowa City, Iowa, US	Neonatologist	
Danielle R.	Rios		MD	University of Iowa Hospitals and Clinics	Iowa City, Iowa, US	Neonatologist	
Robert D.	Roghair		MD	University of Iowa Hospitals and Clinics	Iowa City, Iowa, US	Neonatologist	
Samuel W.	Wong		DO	University of Iowa Hospitals and Clinics	Iowa City, Iowa, US	Neonatologist	
John T.	Wren	Jr	MD, PhD	University of Iowa Hospitals and Clinics	Iowa City, Iowa, US	Neonatologist	
