# Supplementary material for: Outcomes of Infants Born at 21 Weeks’ Gestational Age
Source: JAMA Netw Open. 2025 Dec 12;8(12):e2548211. doi: 10.1001/jamanetworkopen.2025.48211 (PMC12701515; doi:10.1001/jamanetworkopen.2025.48211)
Supplement: Supplement 3. — Data Sharing Statement [file jamanetwopen-e2548211-s003.pdf]

# Data Sharing Statement

Hyland. Outcomes of Infants Born at 21 Weeks' Gestational Age. *JAMA Netw Open*. Published December 12, 2025. doi:10.1001/jamanetworkopen.2025.48211

**Data**
**Data available:** No

**Additional Information**
**Explanation for why data not available:** Protected information
